# Online Pestkoppenstoppen: systematic and theory-based development of a web-based tailored intervention for adolescent cyberbully victims to combat and prevent cyberbullying

**DOI:** 10.1186/1471-2458-14-396

**Published:** 2014-04-24

**Authors:** Niels CL Jacobs, Trijntje Völlink, Francine Dehue, Lilian Lechner

**Affiliations:** 1 Faculty of Psychology, Open University, Valkenburgerweg 177, 6419 AT Heerlen, The Netherlands

**Keywords:** (Cyber) bullying, Intervention, Web-based, E-health, Computer tailoring, Behavior change, Intervention Mapping

## Abstract

**Background:**

The purpose of this article is to give an integrative insight into the theoretical and empirical-based development of the *Online Pestkoppenstoppen* (Stop Bullies Online/Stop Online Bullies). This intervention aims to reduce the number of cyberbully victims and their symptoms of depression and anxiety (program goal), by teaching cyberbully victims how to cope in an adequate and effective manner with cyberbully incidents (program’s outcomes).

**Method/Design:**

In developing the program the different steps of the Intervention Mapping protocol are systematically used. In this article we describe each step of Intervention Mapping. Sources used for the development were a literature review, a Delphi study among experts, focus group interviews with the target group, and elements from a proven effective anti-bullying program. The result is a fully automated web-based tailored intervention for cyberbully victims (12-15 years) consisting of three web-based advice sessions delivered over three months. The first advice aims to teach participants how behavior is influenced by the thoughts they have, how to recognize and dispute irrational thoughts and how to form rational thoughts. In the second advice, participants will learn about the way bullying emerges, how their behavior influences bullying and how they can use effective coping strategies in order to stop (online) bullying. In the third advice, participants receive feedback and will learn how to use the Internet and mobile phones in a safe manner. Each advice is tailored to the participant’s personal characteristics (e.g., personality, self-efficacy, coping strategies used and (ir)rational thoughts). To ensure implementation of the program after testing it for effectiveness, the intervention was pretested in the target-population and an implementation plan was designed. Finally, we will elaborate on the planned randomized controlled trial in which the intervention will be compared to a general information group and waiting list control group. This evaluation will provide insight into the intervention’s efficacy to reduce cyberbullying and its negative effects.

**Discussion:**

Intervention Mapping is a time consuming but profound way to ensure that each step of developing an intervention is taken, and resulted in three web-based tailored pieces of advices that teach adolescents how to cope more effectively with cyberbullying experiences.

**Trial registration:**

NTR3613, 14-09-2012

## Background

The growing popularity of social media (e.g. Facebook, Twitter) and instant messenger services (e.g. IMchat, Twitter) [1] does not always lead to positive experiences. Cyberbullying is defined as a repeated aggressive and intentional act, carried out by a group or an individual, using electronic forms of contact. This act is directed towards a victim who cannot easily defend him or herself [2]. Between 20% and 40% of the adolescents worldwide report being a cyberbully victim [3]. Research has indicated that cybervictimization is associated with serious internalizing difficulties such as depression [4,5], anxiety [6], emotional distress [5] and suicidality [7,8]. Cyberbully victims also more often have experienced drugs, alcohol, physical or sexual abuse, have displayed delinquent and aggressive behavior, have problems at school and have dropped out of school [9-16]. The more adolescents engage in cyber aggression, the more loneliness they feel, the lower their global self-worth is, the fewer mutual friendships they have and the lower their ratings of social acceptance and popularity by peers are [17]. Cyberbullies as well as cyberbully/victims (i.e. both victim and bully) have worse subjective health than those who are not involved [18], and victims of both traditional and cyberbullying are four times more likely to experience depressive symptoms and are five times more likely to attempt suicide compared to non-victims [8].

Unfortunately, intervention programs that deal specifically with cyberbullying are scarce [19]. Moreover, students perceive that methods used in traditional bullying incidents are not equally effective in cyberbullying incidents [20]. Therefore, there is an urgent need for effective cyberbullying interventions. Ideally, these interventions should: (1) do more than increase awareness of potential threats of the Internet by offering victims intensive intervention strategies on the basis of individual needs of the student [21]; (2) offer health education and teach emotional self-management competencies [22]; (3) increase victims´ knowledge of reactive (e.g. deleting, blocking and ignoring messages), preventive (e.g. increased awareness and security) [23] and effective strategies and resources that enable victims to cope with the experienced stress and negative emotions [23]; (4) aim at reducing traditional bullying as well [24], because victims are often involved in both forms of bullying [25-27]; and (5) include training in empathy, Internet etiquette, and healthy Internet behavior [28,29]. Moreover, cyberbully victims often are unwilling to talk to a parent [14], teacher [2,30] or other adults [31]. They spend a lot of time online [32], prefer to get anonymous help [33], and report a need for information and help through the Internet [34]. Therefore, the best method to deliver cyberbullying interventions is via the Internet. Furthermore, web-based interventions can be used whenever and wherever the individual prefers [35], can reach a lot of people in a relatively cheap way [36], and have the possibility to use tailoring [37], which appears to be a successful health promotion technique [38,39].

To increase the effectiveness of such an intervention, a planned, systematic and theory-based approach is needed [40]. Contrastingly, the interventions that currently exist are often based on practical beliefs or commonsense approaches, without a basis in theory or research results [21]. A theoretically sound and evidence-based intervention should provide a description of what works, under what circumstances and for whom [41], with a thorough insight in the relevant determinants of behavioral change, the theoretical methods to change these determinants and how the theoretical methods are translated into practical intervention strategies [42,43]. In order to meet the need for theoretically sound and evidence-based interventions, and based on theory and recommendations from the literature, we have developed an intervention containing three web-based and computer tailored pieces of advice for cyberbully victims: *Online Pestkoppenstoppen* (Stop online bullies/Stop bullies online). This paper focuses on the systematical and theory-based development of the intervention, using the Intervention Mapping (IM) protocol [44]. Effects of the intervention are described elsewhere.

## Method/design

IM is a protocol consisting of six steps that can be used as an iterative process for theory and evidence-based development of health promotion interventions [44]. The six steps include: (1) conducting a needs assessment of the problem, the study population and forming a logic model of the health problem based on the PRECEDE model [45]; (2) defining what program participants have to do in terms of performance objectives, and combining these performance objectives with relevant determinants into change objectives; (3) translating change objectives into practical strategies by selecting theory-based intervention methods; (4) developing, selecting, testing, and producing intervention components in which all strategies are integrated; (5) planning for adoption and implementation of the program; and (6) anticipating on the process and effect evaluation of the program. In this paper we describe how we used each step of the IM protocol to develop the intervention, focusing on the first four steps. We refer to traditional bullying as bullying, to cyberbullying and traditional bullying as (cyber)bullying, and to cyberbully victims and bully/victims as cyberbully victims. Further distinction are provided when necessary.

### Step 1: Needs assessment

The target group for this intervention are adolescents (12-15 years old) from the Continued Secondary Vocational Education who are just starting to attend secondary school, because: (1) cyberbullying appears to occur more frequently in lower levels of secondary education [46-48]; (2) in this period of development the interaction with peers is highly valued and new social networks are formed [49]; and (3) adolescents have an open mind and are eager to learn new skills which enable them to learn how to cope more effectively with problems such as the negative effects of cyberbullying [50].

According to the Transactional Model [51], coping is the cognitive and behavioral effort employed to reduce, master or tolerate internal and external demands resulting from stressful events. In traditional bullying, victims either use more emotion focused [52,53] and passive coping strategies (e.g. crying, expressing emotions) [54-56], or they use emotion focused and aggressive coping strategies (e.g. venting their anger, fighting back). Similarly, cyberbully victims report using aggressive and passive coping strategies [57] (e.g. bullying the bully, deleting messages or pretending to ignore the bullying [58]). Others react to cyberbullying by not retaliating and reacting submissively [59], acting helpless, avoiding the situation [60], doing nothing and displaying avoidant behavior [61]. Some pretend to ignore the bullying, others actually ignore the it [58]. Additionally, adolescents coping with cyberbullying are less likely to seek social support, compared to other strategies [30,53,58,62]. Individuals that are bullied in multiple ways even tend to cope less effectively [63].

It appears that the use of ineffective coping strategies maintains (cyber)bullying [52,54-56,63-67], and that the negative effects of cyberbullying are influenced by the coping style victims use (e.g., ineffective coping appears to yield depression- and health complaints) [68,69]. To increase knowledge about coping strategies used in cyberbullying, it is useful to look at coping strategies employed in cyberbullying and in daily life. These coping strategies appear to not differ significantly, and knowledge about traditional bullying can, to some extent, be applied to cyberbullying [60]. Furthermore, coping strategies used in response to daily stressors are good predictors for coping strategies used in response to cyberbullying [68]. To reduce victimization and its negative effects, it thus appears that adolescents need to improve their current coping strategies. They need to employ effective coping strategies that not only help them to mentally deal with (cyber)bullying, but also contribute to the prevention and discontinuation of (cyber)bullying. Therefore, the primary program goals were reducing the number of: (1) cyberbully victims; and (2) depressive and anxious victims as a consequence of cyberbullying. Secondary goals were a decrease in: (3) victims truanting from school; (4) victims with suicidal thoughts; and (5) an increase in determinants related to cyberbully victims’ behavior, such as self-esteem, self-efficacy, rational helpful beliefs, and effective coping.

### Step 2: Matrices of change objectives

**Figure 1:**
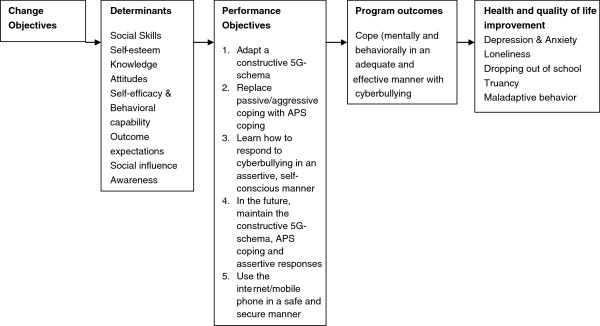
**Change model *Online Pestkoppenstoppen*.** Read figure from right to left to see which steps were taken for the development of the program. Read figure from left to right to see how the program influences behavior and determinants in order to improve health and quality of life.

Next, the focus shifts from the needs assessment to the formation of a change model (Figure 1). In this step intended change in behavior is be further delineated into specific sub-behaviors: the performance objectives (POs). The POs are crossed with relevant determinants in order to create a matrix of change objectives (COs) [44].

#### ***Performance objectives***

First, we translated the risk behavior ineffective coping into a program outcome: after the intervention, cyberbully victims will cope in an adequate and effective manner with (cyber)bullying experiences. This program outcome is a broad conceptualization of what a participant has to do in order to reach the program’s goals. Therefore, participants have to perform specific sub-behaviors, called performance objectives (POs). The POs, and accordingly the content of the intervention, were based on a literature search, focus group interviews with the target group and the linkage group (including the research team, a healthcare coordinator of a school community, a trainer who works with bullied children, and a project leader and director of a website addressing bullied children) formed for this project, a review of relevant (cyber)bullying websites, discussions in an online group about cyberbullying and cybersafety (YouthRiskOnline/Embracecivilty) and a Delphi study among 70 international experts in the field of cyberbullying and coping [70]. In total, eleven performance objectives were formed for this intervention. For an overview of all POs see the first column of Additional file 1.

**Figure 2:**
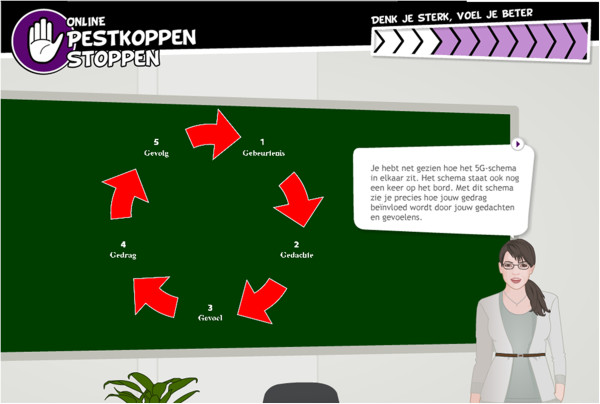
**The 5G-schema in *Online Pestkoppenstoppen. ***The 5G-schema gives insight into the relation between an event (*Gebeurtenis*), a thought (*Gedachte*), a feeling (*Gevoel*), a behavior (*Gedrag*) and a consequence (*Gevolg*).

Based on discussions with the linkage group, it was decided to develop an online translation of the “Pleasure at school” training (PaS) [50]. Victims apparently have negative self-related attitudes, thoughts [71], and self-blaming attributions [72] after a bully experience. PaS is partly based on principles of Rational Emotive (Behavioral) Therapy (RE(B)T) [73,74], and uses the 5G-schema [75] (see Figure 2) to raise awareness in the relation between a thought, feeling and behavior, and to replace irrational thoughts with more rational thoughts. According to RE(B)T, evaluative thoughts mediate the view people have about events that happen and the emotional, behavioral, and inferential reactions to these events [74,76,77]. PaS has proven effective in reducing bullying behavior, anxiety and psychological problems [50] in adolescents. Similarly, RE(B)T has proven effective in the treatment of problems related to cyberbullying [59,78,79] and bullying [4,80], such as anxiety, depression and behavioral problems in adolescents [81,82]. Adolescents therefore learn how to recognize (PO 1), dispute (PO 2) and replace (PO 3) irrational thoughts with rational thoughts.

After thoughts, the 5G-schema focuses on emotions. Relational bullyvictims often have problems with the correct encoding and interpretation of emotions [83]. It is important for an adolescent’s social and cognitive development to correctly perceive and attribute emotions [84]. The understanding and experiencing of emotions of others seems to inhibit antisocial behavior [85], and adolescents who are able to perceive, handle, understand and express their emotions tend to experience better social relationships and are more socially accepted [86]. Furthermore, despite bullyvictims probably being afraid, their most common reactions to victimization are interest and joy [54]. Emotional displays of joy and interest can provide the bully with positive social reinforcement, leading to continued victimization. Therefore, (cyber)bullyvictims need to learn more about emotions and emotion regulation and need to receive health education and emotional self-management competencies [22] (PO 4).

The next aspect the 5G-schema focuses on is behavior. As explained before, whether an adolescent becomes a long-term victim of (cyber)bullying seems to depend on how (s)he copes with (cyber)bullying attempts. Sometimes, the coping strategies employed reduce stress, but the victim fails to confront the bully [70] leading to possible continuation of the bullying. Victims of cyberbullying therefore should become increasingly aware of the ineffectiveness of their current coping style (PO 5). They need knowledge of reactive and preventive strategies (PO 6) and should change their ineffective coping strategies into effective coping strategies (PO 7) [23]. In this paper, all effective coping strategies are named Active Problem Solving (APS) strategies. APS strategies appear to resolve the bullying situation and allows the victim to act assertively [54]. For example, cyberbully victims should try to get help from bystanders, because one quarter of cyberbullying occurs in the presence of witnesses [87]. They should also block or ignore the cyberbully [88], or seek social support [3]. Seeking social support from family is likely to help [89]. Therefore adolescents need information regarding who to ask for help, how to seek help and the benefits of seeking help [88]. Furthermore, having social skills is associated with reductions in victimization and fewer internalizing problems [90]. Cross et al. [91] for example suggested that students should develop their social skills and learn effective ways of addressing relational difficulties online and offline, in order to prevent and reduce cyberbullying. Additionally, interventions should also focus on improving peer relationships in general [88], because social acceptance [92] and integration [93] moderate the impact and consequences of different forms of cyberbullying. Moreover, victims and adolescents not involved in bullying most frequently indicated assertiveness as an intervention strategy [94]. Therefore, interventions should also teach (cyber)bullyvictims non-aggressive responses, and how to cope in an assertive and prosocial manner (PO 8 and 9).

A specific risk factor for cyberbullying is the little control exerted over personal information [27]. A lot of cyberbully victims frequently use the Internet, most of the time in a risky manner [2,78,95]. They share passwords, openly display personal information (e.g. addresses, phone numbers) and communicate with strangers [96,97]. Adolescents should therefore receive cyber safety education, learn to treat each other with respect (both online and offline), and become aware of the importance to regularly change their passwords and never sharing personal information [28] (PO 11).

#### ***Determinants***

The next step in IM is an analysis of the most relevant (i.e. important, changeable) determinants [44] of each PO, by asking what factors determine whether a victim would or is able to perform each PO. The methods used to obtain these determinants were the above mentioned literature review, focus group interviews, a Delphi study on the determinants of ineffective and improved coping with cyberbullying [70], and a study of protocols of (cyber) bullying interventions. Further, several behavior-oriented theories were applied, such as social cognitive theory, theory of planned behavior, transtheoretical model and goal-setting theory. This resulted in a large list of determinants (see the second column of Additional file 1). The aims of this intervention did not include environmental levels (e.g. parents, teachers or schools). Therefore only determinants on the individual level were identified.

#### ***Change objectives***

A matrix was developed to combine performance objectives and their hypothesized determinants into change objectives (COs) [44]. In total, we formed 50 COs. Examples of COs are ‘Individual describes characteristics of irrational and rational thoughts’ and ‘Individual becomes aware of current ineffective (aggressive/passive) coping style’ (see third column Additional file 1). All COs were used in the selection of theoretical methods and applications (step 3) that will change the determinants.

### Step 3: Theory-based methods and practical strategies

**Figure 3:**
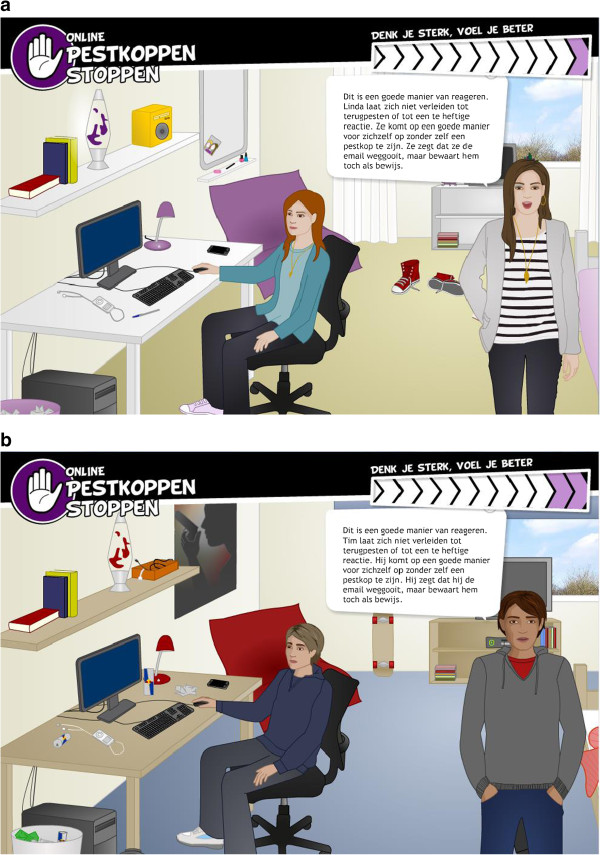
**Example of a model demonstrating behavior. a**. Noa and Linda demonstrating how to act assertively online, and explaining that cyberbullying events (in this case an email) should be saved as evidence. Female participants receive this environment and these digital guides. **b**. Levi and Tim demonstrating how to act assertively online, and explaining that cyberbullying events (in this case an email) should be saved as evidence. Male participants receive this environment and these digital guides.

A theoretical method is a general process that is supposed to influence change in behavioral determinants, based on theories. A practical application is a specific technique for practical use of theoretical methods, in ways that are applicable to the intervention population and context in which the intervention will be used [44]. When a method is translated into a practical application, sufficient understanding of the theory behind the method and its theoretical parameters that determine the effectiveness is needed [98]. For example, to influence determinants such as self-efficacy, modeling [99,100] can be used as a theoretical method. A practical strategy would be to use a role model demonstrating how to react assertively to a difficult situation (see Figure 3a and b). Theoretical parameters for modeling state that modeling is effective when the individual is able to identify with the model, the model demonstrates adequate skills and is reinforced for the behavior displayed [98].

To identify theoretical methods, parameters and practical strategies: (1) the literature was searched for behavior change techniques [44]: (2) the IM book was used to find theoretical methods that influence specific determinant; (3) existing (cyber)bullying interventions were compared; (4) the PaS training [50] was used; and (5) focus group interviews were held, asking the members of the target group what they believed were effective strategies. An overview of theoretical methods and practical applications used in the *Online Pestkoppenstoppen* intervention is shown in the fifth column of Additional file 1. An elucidation of the most important methods follows next.

#### ***Computer tailoring***

As mentioned before, tailoring appears to be a successful health promotion technique [38,39]. Tailoring is the combination of strategies and information intended to reach one specific person by providing personalized feedback based on unique characteristics of a person. These characteristics are assessed individually and are related to the outcome of interest [101]. The use of tailored information results in more improvement in behavior over time compared to the use of generic information [38,102]. Tailored messages are better read, saved and remembered than non-tailored messages [103]. There are two types of tailoring; dynamic tailoring (DT), assessing intervention variables prior to each feedback; and static tailoring (ST), assessing intervention variables at one baseline moment. Although research indicates that DT interventions outperform ST interventions [39], this intervention mostly tailors statical, because several variables used for tailoring are also used for the measurement of effectiveness, and thus need to be assessed prior to each advice. Tailoring is a general technique used throughout the complete intervention and is also combined with the other methods used. Prior to each advice several variables are assessed to enable tailoring. The intervention is for example tailored to personality (ST) as measured by the Big 5 Questionnaire –Short form [104]. Personality may determine a participant’s behavior within a specific context, and this behavior (e.g. reactions, interpretations) can influence the reoccurrence of that event [105]. Other variables on which the intervention provides tailored feedback are for example coping strategies (ST), irrational (ST) and rational (DT) thoughts, self-efficacy (DT), (cyber)bullying experiences (ST) and progress (ST).

#### ***Consciousness raising***

Consciousness raising is used to increase a participant’s awareness about: (1) the thoughts (s)he has after a (cyber)bullying event; (2) his/her own negative 5G-schemas; (3) ineffective coping styles and negative outcomes; and (4) effective coping styles and positive outcomes. Based on answers about irrational thoughts and coping styles, the participant receives tailored feedback concerning his/her irrational thoughts, 5G-schemas, and current coping responses. This feedback may increase awareness, which in turn may help the participant to see why the current way of thinking and/or coping is not helpful or effective. It is important that the participant receives information about problem solving immediately [44]. Therefore, the participant immediately receives information about how to recognize, dispute and replace thoughts after becoming aware of his/her 5G-schema, and how to change ineffective coping into effective coping after becoming aware of his/her ineffective coping.

#### ***Guided practice***

**Figure 4:**
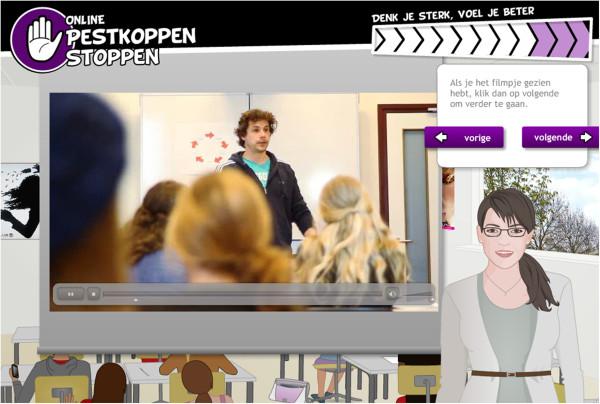
**Video-model explaining/demonstrating skills.** A Dutch actor explains how to recognize, dispute and replace irrational thoughts with rational thoughts, with the help of the 5G-schema.

Guided practice is prompting the participant to rehearse and repeat the desired behavior, discuss the experience and provide feedback [44]. This method is used to increase self-efficacy and behavioral capability. Additionally, it is used to overcome barriers. In this intervention, the participant receives either video-models demonstrating the desired skill (see Figure 4), or comics and digital guides demonstrating and explaining the desired skill, followed by opportunities to practice the skill. Several insight questions are asked in relation to performing the skills, upon which the digital guides give feedback.

#### ***Providing information***

Providing information is used to increase participant’s knowledge, skills, and attitudes, using arguments and persuasive communication [44]. According to the elaboration likelihood model [106], good arguments are effective in reaching stable change in attitudes only when the message is processed through the central route. This happens when the message is personally relevant and not to discrepant from the participant’s beliefs [44]. The participant receives general (e.g. what are the dynamics of a bullying situation, what to do and not to do online) and tailored (e.g. questions that aid recognizing irrational thoughts and changing aggressive/passive coping into active and problem solving coping) information. To check whether the participant remembers the correct information, questions are asked interspersed with feedback. Additionally, to prevent the participant from paying attention to information (s)he already knows, at some points in the intervention (s)he can choose which information (s)he wants to read. Furthermore, all important information is summarized on the personal page and sent to the participant’s e-mail inbox.

#### ***Modeling***

In modeling a person serves as an appropriate model for others that is reinforced for the desired behavior assuming that the behavior of the model will be imitated [107]. Modeling is included in this intervention as a method to increase participant’s self-efficacy and behavioral capability to carry out the desired behavior change, because it is proven to be an effective teaching tool [108]. For example, previous research indicated that video-counseling - in which a real human speaks and asks questions - can be a promising technique for health interventions [109]. In line with the parameters of modeling, the video-models are coping models (i.e. who previously experienced the health problem, but who learned to deal with it effectively), the participant is able to identify with the models, and the models are being reinforced for positive behavior [44]. The short videos were professionally recorded [110]. In several video-clips, models demonstrate and explain how to perform a behavior (e.g. recognizing, disputing and replacing irrational thoughts, coping behaviors).

#### ***Implementation intentions and coping plans***

Research indicates that planning is a powerful self-regulatory tool in the process of behavior change [111,112]. When people plan, they mentally simulate the linking of concrete responses to future situations, replacing spontaneous reactions for pre-planned actions. There are two types of plans: action plans [113], or implementation intentions [114], and coping plans [112]. In action planning, individuals link goal-directed behavior to certain environmental cues by specifying when, where and how to act [112] in if-then statements [115]. As a result, environmental cues should activate - without conscious intent - the initiation of action [112]. In coping planning, individuals anticipate on possible risk situations beforehand by already defining suitable and effective response behavior linked to these potential barriers [112]. Implementation intentions, action and coping plans are found to be effective in translating various intentions into actions [116-118], and appear to be working synergistically in adolescents [119]. In the intervention, the participant makes a combination of implementation intentions and coping plans. A closed-ended plan setting procedure is used to prevent inadequate goals; from a predefined list, the participant chooses the context *if* (either difficult situations or situations in daily life) and action *then* (applications of learned skills, from a predefined list).

### Step 4: Producing program components and materials

Taking the end products of the previous steps into account, we decided to develop one intervention containing three web-based sessions containing tailored pieces of advice addressing mostly personal and psychological determinants. A program plan including the scope, sequence and program materials was developed [44], which is described below. During the process of producing program components and materials, intended program participants and the Linkage group were consulted to determine the preferences for program design by conducting focus group interviews and short voting sessions.

#### ***Scope and sequence***

**Figure 5:**
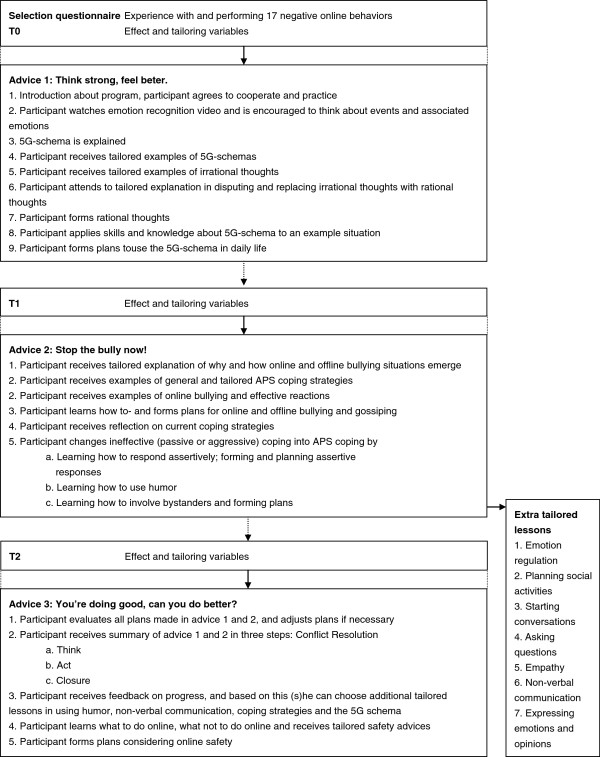
**Flowchart of the web-based tailored advices and questionnaires.** Questionnaires precede each advice, and are used to tailor the advices to the characteristics of each participant. The questionnaires are also used to do the effect evaluation. Each advice consists out of lessons aimed at teaching different skills.

Based on brainstorms and voting sessions with members of the target group, we named the intervention *Online Pestkoppenstoppen* with three web-based tailored pieces of advices named: (1) *Think strong, feel better*; (2) *Stop the bully now!*; and (3) *You are doing great, can you do better?*. Each advice is preceded by several questionnaires – measuring tailoring and effect variables - that take approximately half an hour to complete, and each advice takes approximately 45 minutes to complete. The pieces of advice are offered divided over three months, and e-mails and text messages are used to remind the participant of participation (see Figure 5). The intervention can be found and is accessed on http://www.pestkoppenstoppen.nl.

#### ***Selection***

Because the literature indicates that an explicit approach (e.g. ‘did you experience cyberbullying?’) systematically underestimates the problem [47,58], an implicit approach was used; the individual rates his or her experience with different cyberbullying behaviors. Individuals are included in the intervention if they had experiences with at least one of the cyberbullying behaviors at least ‘once a month’ during the last half year. Cyberbullies and adolescents who did not experience the 17 deviant cyberbehaviors are excluded from participation. Next, the individual (=participant) fills in his or her (nick)name, age, e-mail address, mobile phone number and a login code that is provided to him/her in an information letter. The e-mail address and mobile phone number will be used to remind participants of participation before the start of each advice, during the process of filling in the questionnaires, and during the process of completing each advice.

#### ***Materials***

Below, we shortly describe the different materials developed for the intervention.

#### ***Personal page***

After selection/logging in, the participant is redirected to the personal page, from which (s)he: (1) can proceed or continue with the questionnaires and/or pieces of advice; (2) can find for each advice pictures, video-clips, comics, summaries, formed helpful thoughts, plans and sentences to use; and (3) has access to the extra tailored social skill lessons of advice 2.

#### ***Assessing tailoring and effect measures***

Before receiving each web-based tailored advice, the participant answers several questionnaires (T0, T1 and T2) to assess the effect and tailoring variables. Gender of the participant was used to tailor gender of the guides and contexts (e.g. girls will see Noa, Jessica and a girl’s bedroom, and boys will see Levi, Mike and a boy’s bedroom).

#### ***Advice 1.‘Think strong, feel better.’***

After the baseline measure (T0), the participant starts with the first advice. The participant learns to: (1) work with the 5G-schema in order to recognize (PO 1), dispute (PO 2) and replace (PO 3) irrational thoughts with rational thoughts; (2) carefully examine emotions in response to certain events (PO 4); and (3) form action plans to use the 5G-schema in daily life (PO 10).

#### ***Advice 2 ‘Stop the bully now!’***

A month after the start, the participant begins with the second questionnaire (T1) and advice. The participant: (1) receives knowledge about the dynamics of online and offline bullying situations; (2) receives general APS coping strategies (PO 6); (3) learns and forms implementation intentions for responding towards online and offline bullying (PO 7 and PO 10); (4) becomes aware of ineffective coping style (PO 5); (5) is offered tailored examples of and guidance in APS coping (PO 6 and 7); (6) learns about responding assertively (PO 8 and 9); (7) forms implementation intentions and coping plans regarding assertive responses (PO 10); and (8) receives information about the use and benefits of humor and bystanders (PO 6). Observing victimization on its own can have negative consequences on psychological wellbeing [120]. Therefore, the participant forms implementation intentions and coping plans regarding how to react and involve bystanders in (cyber)bullying situations (PO 10).

#### ***Additional lessons***

Additional social skill lessons (PO 7 and 9) were offered to improve specific skills associated with problem behaviors that are tailored to the participant’s score on several Youth Self-Report (YSR) scales [121]. The participant can choose if, when and which extra lessons (s)he wants to complete. For several extra lessons implementation intentions and coping plans are formed (PO 10). An overview of all lessons tailored to norm scores (above the 93% clinical range on YSR subscales) can be found in Table 1.

**Table 1:** Social lessons based on YSR subscales with norm scores for gender

	**YSR social problems**		**YSR withdrawn/depressed**		**YSR aggression**	
	**Male > 7**	**Female > 7**	**Male > 6**	**Female > 7**	**Male > 12**	**Female > 13**
Starting conversations	X	X	X	X		
Asking questions	X	X	X	X		
Express feelings and opinions	X	X	X	X		
Empathy/social cognition	X	X			X	X
Non-verbal communication	X	X				
Planning to become more socially active			X	X		
Emotional regulation					X	X

Lessons are offered when the participant scores above the 93% clinical range, as can be found in the YSR manual. Norm scores are different for males and females.

#### ***Advice 3 ‘You are doing great, can you do better?’***

Two months after the start, the participant starts with the third questionnaire (T2) and follow up advice. The participant evaluates all plans made in advice 1 and 2 on usability, and if necessary adjusts the plans (PO 10). All skills of advice 1 and 2 are summarized into three steps (i.e. 1. Think; 2. Act; 3.Closure). These three steps together form the skill conflict resolution, which is associated with reductions in victimization and fewer internalizing problems [90]. Tailored on progress, the participant receives feedback (on irrational thoughts, coping responses and YSR scores) and if needed additional lessons (5G-schema, coping styles, humor, non-verbal communication and emotion regulation). Finally, the participant learns and plans how to use the Internet and mobile phone in a safe and secure manner (PO 11).

#### ***Development***

**Figure 6:**
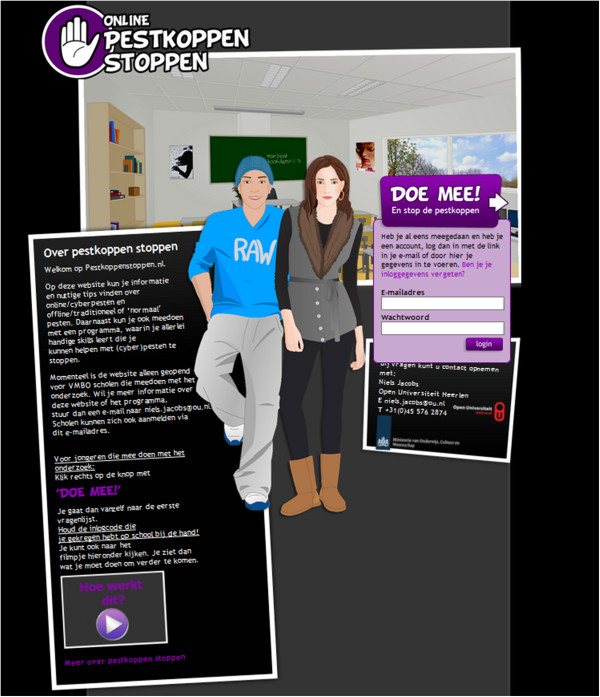
**The website *Online Pestkoppenstoppen.***

The web-based tailored pieces of advice were developed using special software (Nettailor, Evident, the Netherlands), a program that has been developed specifically for web-based tailored interventions, using digital guides in the delivery of the intervention. Research indicates that the integration of an intervention into a website enhances participants’ outcomes and retention [40]. Therefore, the web-based tailored pieces of advice were integrated into a website (http://www.pestkoppenstoppen.nl) (see Figure 6). On this website information about the intervention can be found. After logging in, participants can read general information and watch YouTube video clips about (cyber)bullying, follow the web-based tailored pieces of advice and/or answer the questionnaires.

#### ***Pretest of the interventions***

Parts of the focus group interviews conducted for the development of this intervention were pre- and pilot tests. Members of the target group were instructed to go to the website, complete the questionnaires and follow the pieces of advice. Next, six focus group interviews were held with a total of 41 participants from 4 schools. Based on the results from the pretests, pilot tests and focus group interviews, several adjustments were made: lay-out issues were resolved, bugs were repaired and both the number of questions and the amount of content were reduced.

### Step 5: Adoption and Implementation

A plan for the adoption, implementation and continuation of the web-based tailored intervention was developed. To facilitate adoption and implementation, a linkage group was formed [44]. The intervention was developed in such a way that the potential for adoption, implementation and continuation is enhanced. For example, intervention users can find, register and use the intervention online. Therefore, the implementation of the intervention does not need additional human actions. The only actions needed and costs associated with the implementation, adoption and continuation are recruiting participating schools or individuals, hosting the website, preparing the intervention for implementation after the research has been conducted, and keeping the information up-to-date. Hence, the intervention can easily reach a large number of adolescents and at very low costs.

### Step 6: Planning for evaluation

The evaluation of the program - which is described below - was planned. The research on the effectiveness of the intervention *Online Pestkoppenstoppen* has been approved by the Medical Ethics Committee of the Atrium Hospital of Heerlen (NL39072.096.12; METC nr. 12-T-126), and is registered in the Dutch Trial Register (http://NTR3613).

#### ***Design and procedure***

**Figure 7:**
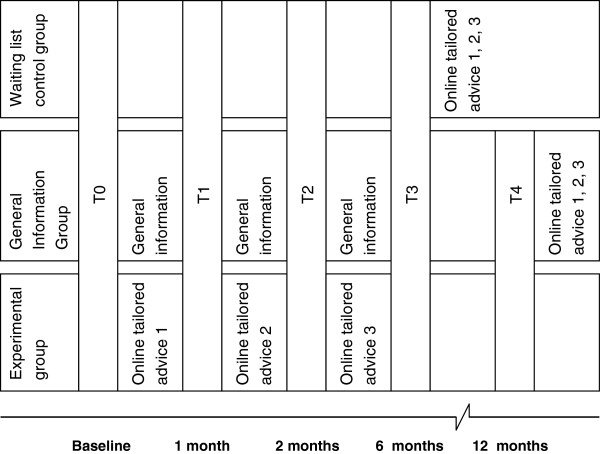
Timing and distribution of the measuring moments between the three conditions.

A three-group (experimental group, general information group and waiting list control group) randomized control trial (RCT) will be conducted to evaluate the efficacy of the intervention, with self-assessed measures at baseline (T0), before advice two (T1), before advice three (T2), a half-year post start (T3) and 1 year post start (T4) (see Figure 7). Statements about the effectiveness of the three tailored web-based pieces of advice compared to general information can be made by comparing the experimental group with the general information group. The waiting list control group allows for controlling internal validation issues such as aging. However, this group does not receive measurements at T4. After the research, both the waiting list control group and general information group will be allowed to use the intervention.

Participants will be recruited via contacting all secondary vocational education schools in the Netherlands. When schools give their consent to participation, randomization will follow. Schools will be stratified on three categories; a) size (small, medium or large); b) location of school (urban or rural); and c) type of education (theoretical or both theoretical and vocational). Schools will be matched and randomly assigned to one of the three intervention conditions. After randomization, all participants will attend a lesson at school in which the research will be explained. Participants receive a written information letter and informed consent, which has to be signed by both the participant and his or her parent(s). Next, participants have to visit the website and complete the selection questionnaire. When a participant meets the inclusion criteria, registration will follow and the research and intervention procedure will start.

A back-up plan was created in case the above-mentioned method of recruiting did not provide a sufficient number of participants. In case of insufficient participants, adolescents are recruited by placing a message on several popular websites often visited by adolescents and/or their parents. Additionally, recruitment messages are sent via Facebook, LinkedIn and Twitter. In this message individual adolescents are recruited to participate in the research by asking them to send us an e-mail with their name, age, name of school and level of education. Next, they receive instructions (written and on video), a written information letter and informed consent for them and their parents. In case parents read the message, they are asked to let their child participate in the research. Only after the adolescent provides us with the signed informed consents from them and their parents, will a login code be sent to the participant with which (s)he can access the intervention and/or research.

#### ***Measurement instruments***

To evaluate the effectiveness of the intervention, several concepts will be measured via self-reported questionnaires. The primary outcomes of this study are frequency of (cyber)bullying, psychological wellbeing, problem behavior, school performance and truancy. These will be assessed at baseline (T0), 6 (T3) and 12 months (T4) follow-up. The secondary outcomes of this study (i.e. moderators or working mechanisms (mediators) of the intervention) are irrational thoughts, self-efficacy, coping with cyberbullying and self-esteem. These will be assessed at baseline (T0), after 1 (T1), 2 (T2), 6 (T3) and 12 months (T4) follow-up. At baseline, several moderators will be measured as well (i.e. demographic characteristics such as gender, age, educational level). In addition, a process evaluation at T3 will be conducted to examine potential moderators.

#### ***Power calculation***

The power analysis is based on two important dependencies: decrease in psychosocial problems and the amount of cyberbullying victims after the intervention. The outcomes are based on the PaS training [122]. A power calculation (effect size = .3; power = .80; intracluster correlation coefficient = .1) showed that at baseline about 446 participants were needed (based on a dropout rate of 20%). Based on previous studies we suspect that 25% of the adolescents between 12 and 15 years can be classified as cyberbullying victims, and that 50% of the adolescents are willing to participate. This results in 3570 adolescents, divided over 30 schools, with each school having 4 or 5 classes.

## Discussion

The aim of this paper was to provide a detailed description of the systematic development of the three web-based tailored pieces of advice for cyberbully victims that aimed at increasing psychological wellbeing, and decreasing victimization, problem behavior, school problems and truancy. In this intervention, cyberbully victims learn how to recognize, dispute and replace irrational thoughts with rational thoughts. Furthermore, their ineffective coping strategies are replaced with effective coping strategies, and they learn how to use the Internet and mobile phone in a safe and secure manner. The intervention is delivered online, via digital guides in digital environments. Digital guides provide information, ask questions, and show videos, pictures, comics and animations.

This intervention has several strengths. First, in developing, implementing and evaluating a systematic approach [44], the Intervention Mapping approach is used. In the process of developing the intervention, empirical evidence, theories, guidelines and recommendations from the cyberbullying and bullying intervention and prevention literature were used to build a solid framework. Second, the intervention aims to change multiple determinants that were found in a study done by Jacobs et al., [70]. Third, guidelines and recommendations from other, not related to (cyber)bullying, intervention studies and theories were used, combined with results from focus groups interviews with the target groups. Fourth, this intervention is tailored to the specific needs of the target group: cyberbully victims are often unwilling to tell a teacher, parent or other adult that they need help. Therefore, they may feel more comfortable to [33] find help anonymously on the Internet [31]. Furthermore, we tried to incorporate all preferences of the target group as indicated in the pre-tests and pilot tests. Fifth, web-based computer tailored interventions can be used any time [35], can reach a lot of people in a relatively cheap way [36], and allow for tailoring [37], which has the potential for a successful health promotion technique [38,39]. Sixth, the planned RCT allows several important conclusions to be drawn. Finally, a process evaluation allows us to draw conclusions about several working mechanisms.

This intervention has a number of limitations. First, it was difficult to reduce the number of questions in the questionnaires we used for evaluation purposes. In the focus group interviews, members of the target group clearly stated that too many questions will lead to drop-out, because answering many questions is experienced as boring and fatiguing. Furthermore, too many questions may cause no-saying or yes-saying and response fatigue (for a review, see [123]), leading to less reliable results. Second, the intervention may contain too much information. Based on the literature, it was decided to include the 5G-schema, coping strategies, social skills, planning and safety instructions. In focus group interviews, members of the target-group mentioned the large amount of information making it difficult to remember everything. Third, we cannot exactly check the degree of usage on the experimental and general information. This may hamper the interpretation of the results. Therefore, we have added process evaluation questions, in which we ask the participants if they have read the information.

In conclusion, the *Online Pestkoppenstoppen* program seems to be a promising start in solving current cyberbullying problems. The results of the evaluation studies, which will be reported in other papers, may contribute to the knowledge about how to stop online victimization and how to use tailoring and web-based interventions in this purpose. Additionally, this intervention may also affect offline victimization, because despite the differences, cyberbullying and traditional bullying also have some overlapping characteristics [24,25,27]. The results can be used as input for other web-based and computer-tailored interventions aimed at cyberbully victims. If the studies point out that the intervention is effective in its purpose, we have an intervention ready for implementation on a large scale.

## Abbreviations

IM: Intervention Mapping; POs: Performance objectives; PaS: Pleasure at school; RE(B)T: Rational Emotive (Behavioral) Therapy; APS: Active Problem Solving; COs: Change Objectives DT, Dynamic Tailoring; ST: Static Tailoring; RCT: Randomized Control Trial; YSR: Youth Self-Report.

## Competing interests

The authors declare that they have no competing interests.

## Authors’ contributions

NJ conceived of the study, developed the online tailored advices, looked for participants, drafted the manuscript and processed all feedback from the other authors. FD and TV helped in brainstorming for- and developing the intervention, looking for participants, helped to draft the manuscript and helped in drafting the final manuscript. LL supervised the whole research and helped with drafting and writing the final manuscript. All authors read and approved the final manuscript.

## Pre-publication history

The pre-publication history for this paper can be accessed here:

http://www.biomedcentral.com/1471-2458/14/396/prepub

## Supplementary Material

Additional file 1: Table S1 Performance objectives, determinants, change objectives, theoretical methods and practical applications of *Online Pestkoppenstoppen. Note*. DG = Digital Guide VM = Video model YSR = Youth Self-Report (Achenbach, [121]) APS = Active, Problem-Solving coping Nvc = non-verbal communication. Click here for file
