# Supplementary material for: Online Pestkoppenstoppen: systematic and theory-based development of a web-based tailored intervention for adolescent cyberbully victims to combat and prevent cyberbullying
Source: BMC Public Health. 2014 Apr 24;14:396. doi: 10.1186/1471-2458-14-396 (PMC4040503; doi:10.1186/1471-2458-14-396)
Supplement: Additional file 1: Table S1 — Performance objectives, determinants, change objectives, theoretical methods and practical applications of Online Pestkoppenstoppen. Note. DG = Digital Guide VM = Video model YSR = Youth Self-Report (Achenbach, [121]) APS = Active, Problem-Solving coping Nvc = non-verbal communication. [file 1471-2458-14-396-S1.pdf]

Table 1

POs, determinants, COs, theoretical methods and practical applications of Online Pestkoppenstoppen

Performance objectives	Determinant	Change objective	Theoretical Method	Practical application
1. Monitor and evaluate thoughts after (cyber)bully experience.	Knowledge	Describe irrational thoughts' differing characteristics.	1. Providing cues<br>2. Tailoring	Participant receives tailored and non-tailored cues from DG that help recognising irrational thoughts.
	Self-efficacy & Behavioural capability	Demonstrate how to recognise thoughts.	1. Providing cues<br>2. Tailoring	VMs demonstrate how to use cues in recognising irrational thoughts and gives participant examples of recognising thoughts, amount of examples tailored to self-efficacy.
	Outcome expectations	Expect to be able to recognise and dispute irrational unhelpful thoughts.	1. Repeated exposure<br>2. Modelling	DG explains and VM demonstrates how to recognise irrational thoughts. Identification tips are provided. Participant is reminded several times

				to be aware of irrational thoughts.
	Awareness	Be aware of irrational thoughts after (cyber)bully experience.	1. Consciousness raising<br>2. Tailoring	DG reflects on tailored irrational thoughts of participant. DG and video model explain the 55G-schema.
2. Gain insight into relationship irrational thoughts, negative emotions and behaviour.	Knowledge	Explain relation between (cyber)bully event, thoughts, feelings, behaviour and consequence.	1. Chunking<br>2. Advanced organisers<br>3. Tailoring	Participant receives explanation (and tailored examples) of (negative) 5G-schemas depicting each G.
	Self-efficacy & Behavioural capability	Demonstrate how to label thoughts (irrational/rational).	1. Guided practice<br>2. Tailoring<br>3. Modelling	DG and video model explain and demonstrate how to label thoughts and give participant the opportunity to practice the labelling of thoughts. Feedback is provided. Amount of practice and example is tailored to

				self-efficacy.
	Awareness	Be aware of relationship irrational thoughts and negative behaviour.	1. Consciousness raising<br>2. Tailoring	Participant receives tailored examples of negative 5G-schemas that are transformed into positive 5G-schemas by changing the irrational thoughts.
3. Replace irrational unhelpful thoughts with rational helpful thoughts.	Knowledge	Define rational thoughts and explain how rational thoughts induce a positive 5G-schema.	1. Persuasive communication<br>2. Chunking<br>3. Tailoring	Participant receives examples of rational thoughts, explanations of why these thoughts will help, and positive 5G-schemas tailored to self-efficacy and coping style.
	Attitude	Feel positive about monitoring, evaluating, disputing and replacing irrational thoughts with rational thoughts.	1. Arguments	VMs explain positive effects after monitoring, evaluating, disputing and replacing irrational thoughts with rational thoughts.

Self-efficacy & Behavioural capability	Demonstrate how to dispute and replace irrational thoughts with rational thoughts.	1. Modelling<br>2. Guided practice<br>3. Direct experiences<br>4. Tailoring	VMs (amount of videos is tailored to self-efficacy) demonstrate disputing and replacing irrational thoughts. Participant and DG practices the replacement of thoughts and receives feedback.
Outcome expectations	Expect to be able to dispute and replace irrational thoughts with rational thoughts.	1. Modelling<br>2. Guided practice<br>3. Repeated exposure<br>4. Active learning	DG explains and VM demonstrates how to replace irrational thoughts with rational thoughts. An overview of replaced thoughts is provided. Rational thoughts should be installed as screensaver prompts, stickers or as a list above the participant's bed. Replacing thoughts is practiced with feedback.

	Self-regulation	Plan to replace thoughts in daily life.	1. Goal-setting	Participant chooses (a) goal(s) related to skills learned in the first advice.
4.Recognise and regulate emotions.	Knowledge	Define emotion regulation strategies.	1. Tailoring<br>2. Persuasive communication	DG explains importance of emotion recognition and regulation. Participant receives emotion regulation techniques tailored to participant's score on the YSR's aggression and/or social problem scale and is encouraged to use them.
	Attitude	Feel positive about regulating emotions.	1. Arguments<br>2. Direct experience<br>3. Tailoring	DG explains to participant with high scores on YSR aggression/social problems why emotion regulation is helpful in (cyber)bullying situations. Participant is encouraged to try the emotion regulation exercises and is

			asked if (s)he feels the relaxation.
Self-efficacy & Behavioural capability	Demonstrate how to recognise and regulate emotions and impulsivity.	1. Guided practice<br>2. Tailoring	Participant with high scores on YSR aggression/social problems watches VM using emotion regulation techniques and receives practice, guidance and feedback in using these techniques. All participants receive models explaining and demonstrating recognition of emotions.
Outcome expectations	Expect that regulating/recognising emotions lead to better outcomes.	1. Direct experience<br>2. Arguments	Participant should experience relaxation and calmness after regulating emotions. VMs provide arguments for the usefulness of recognising emotions.
Self-regulation	Form plans to regulate	1. Active learning	Participant combines sentences into

		emotions in daily life.	2. Implementation intentions	plans for emotion regulation techniques in daily life.
5. Gain insight into ineffective (aggressive/passive) coping style and negative outcomes.	Knowledge	Define ineffective (aggressive/passive) coping strategies and negative outcomes.	1. Tailoring<br>2. Persuasive communication<br>3. Consciousness raising<br>4. Modelling	Participant receives tailored examples of ineffective coping strategies and negative outcomes (in comics, written/spoken text and VMs).
	Awareness	Be aware of current ineffective coping style.	1. Consciousness raising<br>2. Tailoring	DG reflects on participant's current coping strategies and negative outcomes.
6. Gain insight into APS coping and positive outcomes.	Knowledge	Define APS coping strategies and positive outcomes.	1. Persuasive communication<br>2. Advanced organisers	Participant receives examples and overview of tailored and non-tailored APS coping strategies and positive outcomes.
	Attitude	Feel positive about APS	1. Arguments	Positive effects of APS coping are

	coping.	2. Tailoring	explained. Tailored on YSR scores, participant receives instructions and positive effects of different social skills, and is encouraged to practice.
Outcome expectations	Expect positive outcomes associated with APS coping.	1. Direct experience<br>2. Modelling	VM provides and DG explains positive experiences with APS coping. Participant is encouraged to practice the APS skill and think about positive effects.
Social Influence	Explain social influence in APS coping.	1. Information about others' approval	DG describes influence of participant's APS behaviour on other adolescents, what they might think, and the dynamics of a bullying situation.
Awareness	Be aware of positive effects	1. Consciousness raising	DG describes APS coping and

		of APS coping.	2. Framing	positive effects.
7.Replace aggressive/passive coping with APS coping.	Social skills	Demonstrate social skills needed for APS coping.	1. Set tasks on gradient of difficulty<br>2. Tailoring<br>3. Goal setting	Participant receives additional extra social skills lessons tailored on YSR scores. Social skill lessons are linked with plans to use the skills in daily life.
	Self-esteem and self-confidence	Demonstrate and explain importance of self-esteem and self-confidence in APS coping.	1. Persuasive communication<br>2. Modelling	DG explains and importance of, and VMs demonstrate self-esteem/confidence in APS coping.
	Self-efficacy & Behavioural capability	Demonstrate how to use APS coping, apply social cognition and conflict resolution.	1. Set tasks on a gradient of difficulty<br>2. Tailoring<br>3. Enactive mastery experiences	Participant receives APS coping models, explanation and practice in different social skills. Conflict resolution is broken up in sub-skills. Feedback and additional lessons are

			4. Set tasks on a gradient of difficulty	provided tailored to the progress measured.
	Social influence	Recognise importance of seeking social support.	1. Shifting focus	DG lists helpful help-behaviours of peers/parents/teacher when experiencing cyberbullying.
	Self-regulation	Plan to use APS coping strategies.	1. Guided practice<br>2. Modelling<br>3. Persuasion<br>4. Planning coping responses/Implementation intentions	VM demonstrates and DG explains how to form plans, positive effects are provided and participant practices forming own action/coping plans with feedback.
8. Gain insight into assertive behaviour (and nvc patterns*) and its outcomes in relation to	Knowledge	Lists effective nvc patterns.	1. Using Imagery<br>2. Modelling	Participant receives examples of nvc in assertiveness and starting conversations, and is encouraged to feel the difference after applying this

(cyber)bullying.				knowledge.
	Attitude	Feel positive about assertive behaviour.	1. Belief selection<br>2. Persuasive communication	DG explains positive effects of assertive behaviour, participant is encouraged to think about positive effects of assertive behaviour.
	Outcome expectations	Expect positive outcomes associated with assertive behaviour and nvc.	1. Arguments<br>2. Modelling<br>3. Consciousness raising	DG describes positive effects of assertive behaviour. VM and DG demonstrate assertive responding and positive effects. Participant is encouraged to reflect on assertive responding.
9.Replace ineffective (aggressive/passive) behaviour (and nvc*) with	Social skills	Demonstrate social skills needed for assertive behaviour.	1. Set task on a gradient of difficulty	Participant receives explanation and practice in assertive responses in three steps, which can be used online and offline.

effective (assertive) behaviour (and nvc*).	Self-esteem	Demonstrate self-esteem in assertive behaviour and nvc.	1. Persuasive communication<br>2. Modelling	DG and VM explain and demonstrate assertive reactions (with and without assertive nvc*).
	Knowledge	List assertive behaviours.	1. Elaboration<br>2. Use Imagery	Participant receives descriptions of assertive behaviours and how to perform it. Practice is provided by displaying images of “assertive” or strong characters, and participants are encourages to imagine feeling like this character.
	Attitude	Feel positive about adapting assertive behaviour.	1. Belief selection<br>2. Arguments	DG explains positive effects after adapting assertive behaviour, participant is encouraged to think about positive effects of (adapting) assertive behaviour.

Self-efficacy & Behavioural capability	Demonstrate when and how to display assertive behaviour and nvc.	1. Guided practice<br>2. Repeated exposure<br>3. Tailoring	Participant receives VMs and comics demonstrating assertive behaviour tailored to coping style measured.
Social influence	Recognise social influence in assertive behaviour and nvc.	1. Shifting focus	DG acknowledges that others' presence can have influence on responding in an assertive manner.
Awareness	Be aware of positive effects associated with assertive behaviour.	1. Consciousness raising<br>2. Framing	DG explains that assertive reactions lead to positive outcomes. Participant is encouraged to think about the lessons they have learned.
Self-regulation	Plan to use assertive reactions and behaviour.	1. Planning coping responses<br>2. Implementation intentions	Participant first forms assertive responses from predefined lists. Next, participant forms coping plans with these assertive responses, choosing

				from predefined lists.
10.Maintain the constructive 5G-schema, APS coping and assertive responses.	Attitude	Feel committed to maintaining constructive 5G-schema, APS coping and assertive behaviour.	1. Arguments<br>2. Tailoring	DG encourages participant to practice all lessons in daily life, with warnings for possible failure, but with the message “Do not give up, practice makes perfect”. Feedback is provided about progress.
	Self-efficacy & Behavioural capability	Demonstrate how to maintain constructive 5G-schema, APS coping and assertive behaviour.	1. Goal setting<br>2. Planning coping responses	Participant forms plans linking APS to daily life (difficult) situations, aided by DG.
	Outcome expectations	Expect positive outcomes after	1. Persuasive communication	DG explains positive effects of APS coping and stresses the importance of

		maintaining constructive 5G-schema, APS coping and assertive behaviour.		keep using this coping strategy.
	Self-regulation	Evaluate and/or adjust plans to use the skills learned concerning 5G-schema, APS coping and assertive responding lessons.	1. Tailoring 2. Active learning 3. Implementation intentions	Participant evaluates own plans, and adjusts if necessary.
11.Prevent and solve negative cyber bully experiences by using the Internet and mobile phones in a safe and secure manner.	Knowledge	List security and safety advices for the Internet and mobile phones.	1. Prompts	Participant receives prompts concerning safe Internet/mobile phone use.
	Attitude	Feel positive about safe and secure use of the Internet and mobile phones.	1. Arguments	DG explains positive effects of safety tips and guidelines safe Internet and mobile phone use.

Self-efficacy & Behavioural capability	Demonstrate how to use the Internet and mobile phones in a secure manner.	1. Modelling	DG explains and demonstrates (via screenshots) how to form safe passwords, and provides information how to protect yourself on the Internet.
Outcome expectations	Expect positive outcomes after using the Internet and mobile phones in a secure manner.	1. Arguments	DG explains participant that safe use of the Internet and mobile phone leads to less cyberbullying.
Awareness	Become aware of own insecure and unsafe use of the Internet and mobile phones.	1. Consciousness raising<br>2. Loss frame	DG explains that unsafe internet use heightens the chance of becoming a cyberbully victim. Participant is encouraged to think about own Internet-behaviour
Self-regulation	Form plans to keep using	1. Goal setting	Participant chooses plans related to

	the Internet in a secure manner	2. Implementation intentions	the safe use of Internet and resetting passwords regularly.

Note. DG= Digital Guide

VM= Video model

YSR= Youth Self-Report (Achenbach, 1991)

APS= Active, Problem-Solving coping

Nvc= non-verbal communication
